# Instrumented Assessment of Gait in Pediatric Cancer Survivors: Identifying Functional Impairments After Oncological Treatment—A Pilot Study

**DOI:** 10.3390/children13010096

**Published:** 2026-01-09

**Authors:** María Carratalá-Tejada, Diego Fernández-Vázquez, Víctor Navarro-López, Juan Aboitiz-Cantalapiedra, Francisco Molina-Rueda, Blanca López-Ibor Aliño, Alicia Cuesta-Gómez

**Affiliations:** 1 Physical Therapy, Occupational Therapy, Rehabilitation and Physical Medicine Department, Faculty of Health Sciences, Rey Juan Carlos University, 28922 Madrid, Spain; maria.carratala@urjc.es (M.C.-T.); victor.navarro@urjc.es (V.N.-L.); juan.aboitiz@urjc.es (J.A.-C.); francisco.molina@urjc.es (F.M.-R.); alicia.cuesta@urjc.es (A.C.-G.); 2 Movement Analysis, Biomechanics, Ergonomics, and Motor Control Laboratory, Faculty of Health Sciences, Rey Juan Carlos University, 28922 Madrid, Spain; 3 Pediatric Hematology and Oncology Unit, Hospital HM Montepríncipe, 28660 Madrid, Spain; blopezibor@hmhospitales.com; 4 Hospital Universitario Fundación Alcorcón, 28922 Madrid, Spain

**Keywords:** gait, biomechanics, cancer, chemotherapy-induced peripheral neuropathy, children, statistical parametric mapping

## Abstract

**Highlights:**

**What are the main findings?**

- Pediatric cancer survivors exhibit significant gait alterations compared with healthy peers, including slower walking speed, pelvic instability, reduced hip extension, knee hyperextension, and diminished ankle plantarflexor moments.
- These biomechanical deviations reflect persistent neuromuscular impairments likely related to chemotherapy-induced neurotoxicity and treatment-related deconditioning.

**What are the implications of the main findings?**

- Instrumented 3D gait analysis provides an objective method to detect subtle locomotor deficits in childhood cancer survivors.
- Early identification of these abnormalities can guide targeted rehabilitation strategies to enhance long-term functional outcomes and quality of life.

**Abstract:**

**Background/Objectives**: Pediatric cancer survivors frequently experience neuromuscular sequelae related to chemotherapy-induced neurotoxicity. Agents such as vincristine, methotrexate, and platinum compounds can lead to persistent gait alterations and sensorimotor deficits that impair mobility and quality of life. This study aimed to objectively assess gait in pediatric cancer survivors after the completion of oncological pharmacological treatment to identify specific spatiotemporal, kinematic, and kinetic alterations and characterize neuromechanical patterns associated with neurotoxic exposure. **Methods**: A cross-sectional observational study was conducted including pediatric cancer survivors (6–18 years) who had completed chemotherapy and age- and sex-matched healthy controls. Gait was analyzed using a Vicon^®^3D motion capture system, with reflective markers placed on standardized anatomical landmarks. Spatiotemporal, kinematic, and kinetic variables were compared between groups using parametric tests and statistical parametric mapping (SPM) with Holm–Bonferroni correction (α = 0.05). **Results**: Pediatric cancer survivors showed slower gait velocity (Mean Difference (MD) = 0.17, *p* = 0.018, Confidence Interval CI95% = 0.04; 0.4), shorter step (MD = 0.1, *p* = 0.015, CI95% = 0.01; 0.19) and stride length (MD = 0.17, *p* = 0.018, CI95% = 0.03; 0.31), as well as reduced single support time (MD = 0.1, *p* = 0.043, CI95% = 0.01; 0.19), along with significant alterations in pelvic, hip, knee, and ankle kinematics compared with controls. Increased pelvic elevation (MD = 0.92, *p* = 0.018, CI95% = 0.25; 1.58), reduced hip extension during stance (MD = −2.99, *p* = 0.039, CI95% = −5.19; −0.74), knee hyperextension in mid-stance (MD = −3.84, *p* < 0.001, CI95% = −6.18; −0.72), and limited ankle dorsiflexion (MAS MD = −4.04, *p* < 0.001, CI95% = −6.79; −0.86, LAS MD = −3.16, *p* < 0.001) and plantarflexor moments in terminal stance (MAS MD = −149.65, *p* = 0.018, CI95% = −259.35; −48.25, LAS MD = −191.81, *p* = 0.008, CI95% = −323.81; −57.31) were observed. Ground reaction force peaks during loading response (MAS MD = −16.86, *p* < 0.001, CI95% = −26.12; −0.72 LAS MD = −11.74, *p* = 0.001, CI95% = −19.68; −3.94) and foot-off (MAS MD = 10.38, *p* = 0.015, CI95% = 0.41; 20.53, LAS MD = 11.88, *p* = 0.01, CI95% = 3.15; 22.38) were also reduced. **Conclusions**: Children who have completed chemotherapy present measurable gait deviations reflecting persistent neuromechanical impairment, likely linked to chemotherapy-induced neurotoxicity and deconditioning. Instrumented gait analysis allows early detection of these alterations and may support the design of targeted rehabilitation strategies to optimize functional recovery and long-term quality of life in pediatric cancer survivors.

## 1. Introduction

Pediatric oncology encompasses a wide range of neoplastic conditions, both hematologic and solid, characterized by uncontrolled cellular proliferation [1]. Although childhood cancers represent only 1–3% of all malignancies, they remain a leading cause of disease-related mortality among children and adolescents under the age of 18 [2]. According to the World Health Organization, an estimated 400,000 new cases of cancer occur annually in individuals under 20 years of age, though many go undiagnosed, especially in low- and middle-income countries [3]. While survival rates in high-income countries exceed 80%, disparities in access to care result in survival rates as low as 10–20% in lower-income regions [4,5].

In Spain, the RETI-SEHOP registry estimates an incidence of 1500 pediatric cancer cases annually, with five-year survival rates reaching 82% among children aged 0–14 [6,7]. These improvements in survival have redirected clinical and scientific efforts toward understanding long-term treatment-related complications.

Chemotherapy-induced peripheral neuropathy (CIPN) and central neurotoxicity have emerged as significant long-term sequelae in survivors of childhood cancer [8,9,10]. Neurotoxic agents such as vincristine, platinum derivatives, methotrexate, and corticosteroids are widely used in frontline pediatric oncology protocols [8,9,11], with a growing body of evidence showing that such agents can lead to sensorimotor deficits that persist for months or even years after treatment cessation [10,12]. These may manifest as gait disturbances, balance impairments, reduced muscle strength, fatigue, and pain, ultimately affecting mobility, autonomy, and quality of life [12,13,14].

Children are particularly vulnerable to these toxic effects due to the ongoing development of their nervous and musculoskeletal systems [9,13]. Several studies have reported limitations in ankle dorsiflexion, alterations in gait kinematics, reduced walking speed, and abnormalities in muscle activation patterns in pediatric cancer survivors [13,14,15,16]. These neuromechanical alterations are associated with decreased functional efficiency, social participation restrictions, and increased risk of secondary complications such as sedentary behavior or deconditioning [14,17].

Despite this, there is a lack of systematic evaluations of gait using advanced biomechanical tools in this population. Objective assessments, such as 3D gait analysis, can provide quantitative insights into the extent of sensorimotor impairment and help define neurofunctional phenotypes associated with specific treatment exposures [15,16,17,18].

Few studies have employed gait analysis techniques to characterize motor impairments in pediatric cancer survivors, revealing alterations in spatiotemporal, kinematic, and kinetic parameters associated with treatment-related neurotoxicity [13,14,15,16]. However, most available evidence comes from international cohorts, and comprehensive biomechanical assessments in Spanish pediatric populations remain scarce. The use of three-dimensional gait analysis provides objective and quantitative information that can support clinicians in detecting subtle gait abnormalities and tailoring rehabilitation strategies. Early identification of persistent neuromotor deficits may enable timely intervention and contribute to optimizing long-term functional outcomes and quality of life [17,18,19].

Although international studies have described gait deviations in pediatric cancer survivors, the evidence remains limited, heterogeneous, and primarily focused on specific treatments or small samples. Importantly, no comprehensive, instrumented 3D gait analyses have been conducted in pediatric cancer survivors within Spain, despite differences in treatment protocols, rehabilitation pathways, and survivorship care models that may influence motor outcomes. Furthermore, to our knowledge, no previous studies have examined detailed spatiotemporal, kinematic, and kinetic gait parameters using a gold-standard optoelectronic motion capture system in this population in the Spanish context. This lack of population-specific biomechanical data restricts clinicians’ ability to identify characteristic neuromechanical patterns and to design targeted rehabilitation strategies tailored to Spanish pediatric oncology survivors.

This study aims to comprehensively evaluate gait in pediatric patients after completing pharmacological cancer treatment. Using a gold-standard three-dimensional optoelectronic gait analysis system, it seeks to identify the main spatiotemporal, kinematic, and kinetic alterations [20]. By characterizing the most affected variables and neuromechanical patterns potentially associated with neurotoxic treatment exposure, this study intends to contribute to early detection, targeted rehabilitation, and the optimization of functional recovery in childhood cancer survivors. Given the limited availability of Spanish pediatric oncology survivors and the exploratory nature of the present work, this study should be considered a pilot study, intended to provide preliminary biomechanical evidence and to guide future research with larger and more age-specific cohorts.

## 2. Materials and Methods

### 2.1. Desing

A cross-sectional observational study was conducted involving the voluntary participation of individuals diagnosed with a neoplastic condition and healthy control subjects. The protocol received approval from the Ethics Committee of the Rey Juan Carlos University (approval internal code: 060720211662), and all participants provided written informed consent (or their parents) prior to enrollment. The research procedures followed the recommendations outlined in the STROBE guidelines.

### 2.2. Participants

All study procedures complied with the ethical standards set forth in the Declaration of Helsinki and Law 14/2007, of 3 July, on Biomedical Research. Participation of both individuals with pediatric cancer and healthy controls was entirely voluntary. Recruitment was conducted between January 2022 and June 2023. Patients were referred from specialized pediatric oncology units at HM Hospitals in the Community of Madrid by medical staff and physiotherapists once inclusion criteria were met. Eligible participants were children and adolescents aged 6 to 18 years, diagnosed with either solid or hematologic neoplasms, who had completed pharmacological treatment protocols involving high-dose corticosteroids and/or neurotoxic chemotherapy agents. Exclusion criteria included intolerance to study procedures, tumors located in the central nervous system, history of limb amputation or reconstructive surgery, radiotherapy affecting the musculoskeletal system (especially lower limbs and pelvis), and sensory impairments (vision or hearing). Participants attended the Movement Analysis, Biomechanics, Ergonomics, and Motor Control Laboratory (LAMBECOM). The control group, matched by age and sex, consisted of healthy children and adolescents without musculoskeletal, neurological, psychiatric, or cardiovascular conditions affecting posture or mobility, and were recruited among relatives or acquaintances of university staff.

### 2.3. Experimental Protocol

The determination of the most affected side (MAS) and the least affected side (LAS) was performed through medical history and clinical examination. Specifically, each patient was asked which side they perceived as having the greatest functional impairment, and this was subsequently confirmed through physical evaluation. This combined information was used to establish which side was most affected in each case.

For the instrumental gait analysis, the gold-standard system used was a Vicon^®^ Vero 2.2 ™ motion capture system with eight cameras operating at 330 Hz, synchronized with three floor-mounted AMTI^®^ force plates (Watertown, MA, USA, 1000 Hz) via Vicon Nexus™ 2.15 software. Reflective markers were placed according to the Vicon^®^ Lower-Body Plug-in Gait model [21].

After instrumentation, participants were instructed to walk along a corridor approximately 11 m in length at a self-selected, comfortable pace. For each session, ten gait trials were recorded per individual.

Data processing was performed using Vicon^®^ Nexus software version 2.15, applying the Vicon^®^ Lower-Body Plug-in Gait model. This process included the reconstruction of marker trajectories, labeling, and filtering of biomechanical data.

To minimize the risk of bias, the individual responsible for instrumenting the subjects, the evaluator processing the recordings, and the researcher conducting the statistical analysis operated independently from each other. Both study groups underwent the same experimental protocol. All measurements were conducted LAMBECOM, Faculty of Health Sciences, Rey Juan Carlos University.

### 2.4. Outcome Measures

Spatiotemporal gait parameters, joint kinematics and kinetics were derived from the average of ten complete gait cycles recorded for each participant. Comparisons were made between the non-dominant side of healthy controls and the MAS of patients, as well as between the dominant side and the LAS. The spatiotemporal parameters assessed included gait velocity (m/s), stride length (m), step width (m), cadence (steps/min), and the timing of foot-off within the gait cycle (GC) (expressed as a percentage). Kinematic variables were evaluated in three planes of motion—sagittal, frontal and horizontal—for the pelvis and hip, and exclusively in the sagittal plane for the knee and ankle. Joint internal moments were evaluated in three planes of motion for the hip, exclusively in the sagittal plane for the knee and ankle, and the normalized ground reaction force (GRF) in vertical plane.

### 2.5. Data Analysis

Spatiotemporal parameters were analyzed using either a parametric independent two-sample t-test or the non-parametric Mann–Whitney U test, as appropriate, whereas gait kinematics and kinetics were evaluated through the multivariate statistical parametric mapping (SPM) Hotelling’s T^2^ test, followed by univariate SPM *t*-tests for each joint. For all analyses, SPM{T^2^} or SPM{t} statistics were computed, together with the corresponding thresholds for rejection of the null hypothesis, maintaining a false-positive error rate of 0.05. The family-wise error rate for both SPM{T^2^} and SPM{t} tests was determined for each joint, and the Holm–Bonferroni correction was applied to ensure that the per-family error rate of the SPM *t*-tests remained below 0.05.

Effect sizes were calculated using Hedges’ g for parametric tests and Cohen’s r was reported for non-parametric tests to quantify the magnitude of the effect (results are presented in the Supplementary Material).

### 2.6. Sample Size Estimation

No priori sample size calculation was performed due to the limited accessibility of this clinical population and the practical recruitment constraints inherent to pediatric oncology. This limitation is consistent with previously instrumented gait research in childhood cancer survivors, which has typically involved modest sample sizes. For example, studies by Wright et al. [16] and Beulertz et al. [13] reported sample sizes ranging from 13 to 17 participants and still identified clinically meaningful gait alterations. These precedents highlight the common recruitment challenges in this field and align with the constraints encountered in the present study.

## 3. Results

**Figure 1:**
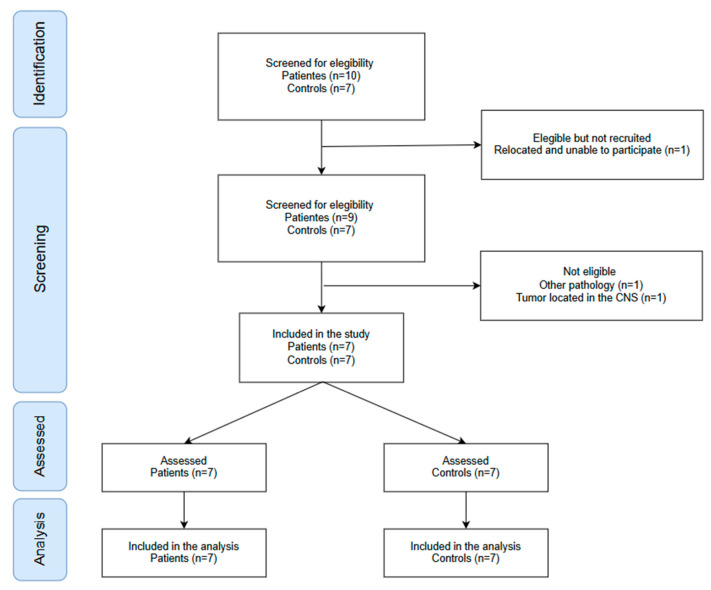
Flow diagram of participant recruitment.

A total of 14 participants were included in the study, evenly distributed between the control (n = 7) and experimental (n = 7) groups. Figure 1 summarizes the process. Both groups showed a similar sex distribution, with five females and two males in each group. No significant differences were observed between groups in age (*p* = 0.637), weight (*p* = 0.948), or height (*p* = 0.972). All participants in the experimental group were pediatric cancer survivors, including cases of Ewing sarcoma (n = 3, 42.86%), leukemia (n = 1, 14.29%), lymphoma (n = 1, 14.29%) and rhabdomyosarcoma (n = 2, 28.57%). The treatment protocols used COG AEWS 1031 (n = 3, 42.86%), LLA-SHOP/PETHEMA (n = 1, 14.29%), EpSSG RMS2005 (n = 1, 14.29%), and COG ARST0431 (n = 2, 28.57%). The mean duration of treatment was 195.71 (135.38) days. All participants were evaluated between 15 and 30 days after completing the treatment.

### 3.1. Spatiotemporal Parameters

Significant differences were observed between groups in several spatiotemporal gait parameters (Table 1). The control group showed a higher walking speed compared with the experimental group (1.37 (0.16) m/s vs. 1.15 (0.14) m/s; *p* = 0.017), as well as greater step length (0.69 (0.07) m vs. 0.59 (0.07) m; *p* = 0.026) and stride length (1.39 (0.14) m vs. 1.22 (0.09) m; *p* = 0.038). A significant difference was also found in single support time, which was longer in the control group (0.42 (0.03) s vs. 0.32 (0.11) s; *p* = 0.043). No significant differences were observed between groups in cadence, step time, step width, double support time, or in specific gait cycle events (foot-off and opposite foot contact).

### 3.2. Kinematics

**Figure 2:**
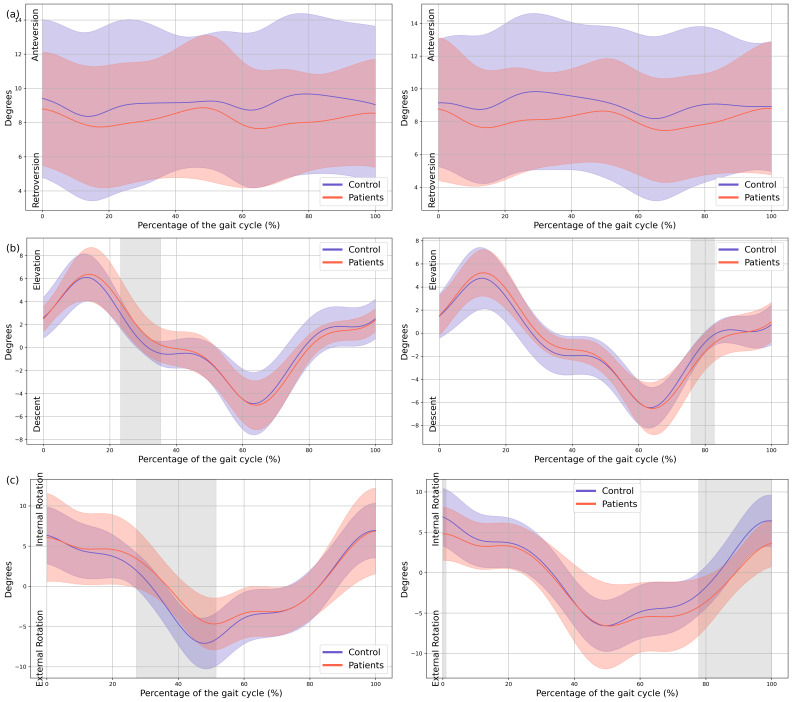
**Pelvic kinematics.** (**a**) Sagittal plane; (**b**) Frontal plane; (**c**) Transverse plane. The *X*-axis represents the normalized gait cycle percentage from 0 to 100, and the *Y*-axis represents the angle in degrees. The left panels compare the dominant side of healthy controls (blue line) with the less affected side of the patients (red line), and the right panels compare the non-dominant side (blue line) with the more affected side (red line). The red and blue band indicates the standard deviation of the patients and healthy controls respectively. The gray band indicates the percentage of the gait cycle in which statistically significant differences between groups were observed.

In the patient group, a significant upward displacement of the MAS hemipelvis was observed in the frontal plane during mid-stance (23–35% GC, Mean Difference (MD) = 0.92 *p* = 0.018, Confidence Interval (CI) = 0.25; 1.58), this pelvic movement corresponds with the descent of the LAS hemipelvis throughout the swing phase (75–82% GC, MD = −0.78, *p* = 0.037, CI = −1.31; −0.2). There is also an internal rotation of the MAS hemipelvis in the transverse plane during mid stance (27–51% GC, MD = 2,52, *p* = 0.003, CI = 0.36; 4.31), which coincided with an external rotation of the LAS hemipelvis during terminal swing (77–100% GC, MD = −2.63, *p* = 0.006, CI = −4.37; −0.43) (Figure 2).

**Figure 3:**
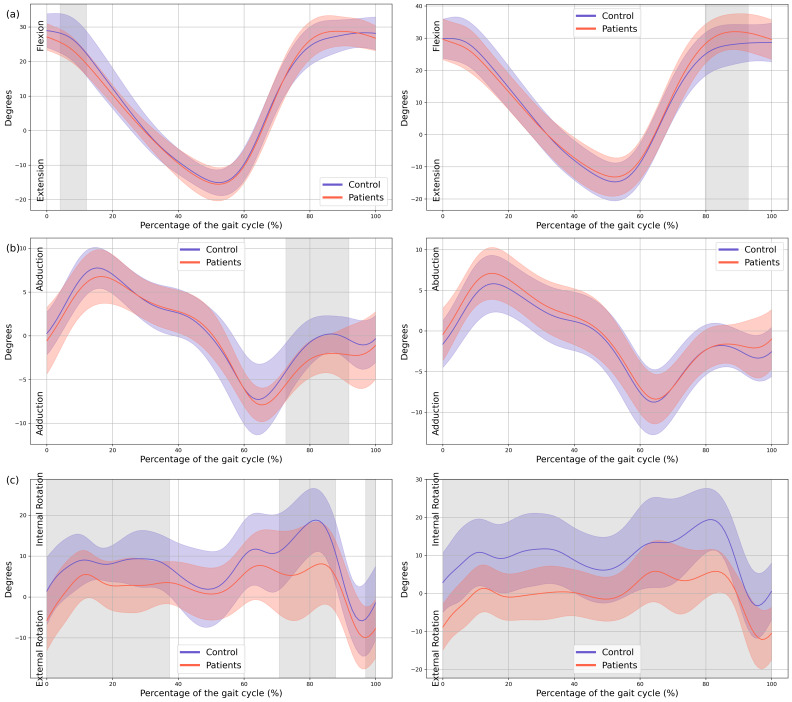
**Hip kinematics.** (**a**) Sagittal plane; (**b**) Frontal plane; (**c**) Transverse plane. The *X*-axis represents the normalized gait cycle percentage from 0 to 100, and the *Y*-axis represents the angle in degrees. The left panels compare the dominant side of the healthy controls (blue line) with the less affected side of the patients (red line), and the right panels compare the non-dominant side (blue line) with the more affected side (red line). The red and blue band indicates the standard deviation of the patients and healthy controls respectively. The gray band indicates the percentage of the gait cycle in which statistically significant differences between groups were observed.

In the patient group, a significant decrease in MAS hip extension was observed during the loading response phase (4–12% GC, MD = −2.99 *p* = 0.039, CI = −5.19; −0.74), coinciding with an increased LAS hip flexion during terminal swing (79–92% GC, MD = 3.69, *p* = 0.039, CI = 0.75; 6.36). Additionally, greater MAS hip abduction in the frontal plane was noted during swing (72–91% GC, MD = −1.94, *p* = 0.005, CI = −3.11; −0.43), together with an overall increase in external rotation of both hips throughout the GC (MAS 0–37% GC, MD = −5.41, *p* < 0.001, CI = −10.16; −1.29, 70–88% GC, MD = −8.89, *p* = 0.002, CI = −14.91; −1.42 and 96–100% GC, MD = −5.44, *p* = 0.047, CI = −9.45; −1.31, LAS 0–100% GC, MD = −9.91, *p* < 0.001, CI = −17.07; −3.56) (Figure 3).

**Figure 4:**
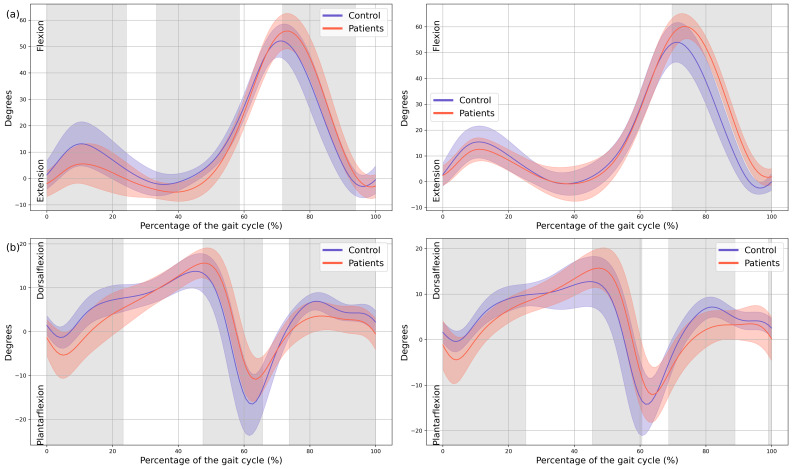
**Knee and ankle kinematics.** (**a**) Sagittal plane of the knee; (**b**) Sagittal plane of the ankle. The *X*-axis represents the normalized gait cycle percentage from 0 to 100, and the *Y*-axis represents the angle in degrees. The left panels compare the dominant side of the healthy controls (blue line) with the less affected side of the patients (red line), and the right panels compare the non-dominant side (blue line) with the more affected side (red line). The red and blue band indicates the standard deviation of the patients and healthy controls respectively. The gray band indicates the percentage of the gait cycle in which statistically significant differences between groups were observed.

Regarding the knees, the MAS knee of the patient group showed a significant reduction in knee flexion during loading response (0–24% GC, MD = −5.81, *p* < 0.001, CI = −10.69; −1.21), hyperextension during mid-stance (33–58% GC, MD = −3.84, *p* < 0.001, CI = −6.18; −0.72), and increased flexion during mid-swing (71–94% GC, MD = 6.8, *p* < 0.001, CI = 0.87; 12.98). In the LAS increased knee flexion was observed during both mid- and terminal swing phases (70–100% GC, MD = 9.32, *p* < 0.001, CI = 0.7; 16.84) (Figure 4).

Significant changes were also observed in both ankles. Increased plantarflexion was noted during heel strike and loading response, accompanied by reduced dorsiflexion at the beginning of mid-stance (MAS 0–23% GC, MD = −4.04, *p* < 0.001, CI = −6.8; −0.86, LAS 0–25% GC, MD = −3.16, *p* < 0.001, CI = −5.52; −0.57). During terminal stance, dorsiflexion increased, whereas plantarflexion decreased during pre-swing (MAS 47–65% GC, MD = 5.66, *p* < 0.001, CI = 0.74; 11.17, LAS 45–60% GC, MD = 5.46, *p =* 0.002, CI = 0.82; 11.15), and dorsiflexion was reduced throughout the swing phase (MAS 73–100%, MD = −2.4, *p* < 0.001, CI = −4.55; −0.54, LAS 69–89% GC, MD = −3.54, *p* < 0.001, CI = −5.95; −0.49) (Figure 3).

### 3.3. Kinetics

**Figure 5:**
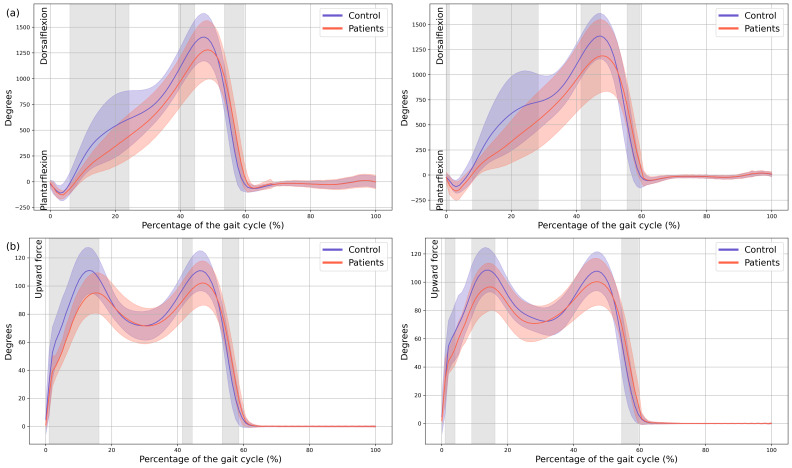
**Kinetics.** (**a**) Ankle internal moment, where positive values indicate an internal plantarflexion moment; (**b**) Ground reaction force (GRF), where positive values indicate upward force. The *X*-axis represents the normalized gait cycle percentage from 0 to 100, and the *Y*-axis represents the magnitude of the kinetic variable in standard units. The left panels compare the dominant side of the healthy controls (blue line) with the less affected side of the patients (red line), and the right panels compare the non-dominant side (blue line) with the more affected side (red line). The red and blue band indicates the standard deviation of the patients and healthy controls respectively. The gray band indicates the percentage of the gait cycle in which statistically significant differences between groups were observed.

A significant reduction in plantarflexor was observed in both feet of the patients during terminal stance moment (MAS 39–44% GC, MD = −149.65, *p* = 0.018, CI = −259.35; −48.25, LAS 41–48% GC, MD = −191.81, *p* = 0.008, CI = −323.81; −57.31). Additionally, the first peak of the ground reaction force corresponding to loading response was reduced in both lower limbs of the patients (MAS 1–15% GC, MD = −16.86, *p* < 0.001, CI = −26.12; −0.72, LAS 9–16% GC, MD = −11.74, *p* = 0.001, CI = −19.68; −3.94) and in foot-off (MAS 54–59% GC, MD = 10.38, *p* = 0.015, CI = 0.41; 20.53, LAS 55–60% GC, MD = 11.88, *p* = 0.01, CI = 3.15; 22.38) (Figure 5).

Appendix A presents all graphs along with their corresponding SPM statistics, the established threshold, and the *p*-values for the segments exceeding the threshold, indicating statistically significant differences.

## 4. Discussion

This study provides a detailed biomechanical characterization of gait in young pediatric cancer survivors after chemotherapy, revealing significant spatiotemporal alterations compared to healthy controls. Participants with a history of oncological treatment showed slower walking speed, shorter step and stride length, and reduced single support time. These findings may suggest the presence of residual neuromechanical impairments that persist after the completion of chemotherapy, likely reflecting possible impacts of neurotoxic treatments on locomotor function during the early post-treatment phase.

Regarding the spatiotemporal parameters, the lower gait speed and reduced step and stride length observed in the pediatric cancer survivors indicate decreased gait efficiency and propulsion capacity, which may reflect mild motor control alterations; notably, these differences were accompanied by large effect sizes, underscoring the robustness and clinical relevance of these findings. This agrees with previous studies showing that children and adolescents treated for acute lymphoblastic leukemia (ALL) walk more slowly, with shorter steps and lower cadence than healthy controls, likely as a compensatory strategy to enhance stability [13,14,15,17,19]. Recent evidence supports these findings: Gilchrist et al. [14] and Wright et al. [16] reported that pediatric cancer survivors with CIPN walked with reduced speed and step length, associated with decreased ankle power, and altered muscle activation patterns. Likewise, Antunes et al. (2024) [22] observed in children undergoing ALL maintenance therapy that gait velocity, cadence, and step length were significantly lower than normative values. Such reductions in gait dynamics may be related to both central and peripheral mechanisms. Chemotherapy agents such as vincristine, platinum compounds, and methotrexate are known to cause axonal neuropathy, proprioceptive deficits, and distal weakness [8,23,24]. These neurotoxic effects could contribute to disrupting sensorimotor integration, leading survivors to adopt slower and less efficient gait possibly as a stabilization strategy, at the cost of energetic efficiency.

The kinematic analysis provided a detailed view of lower-limb movement patterns, revealing several neuromechanical alterations in pediatric cancer survivors. At pelvis level, participants exhibited frontal-plane instability, including increased contralateral pelvic drop and significant elevation of the stance hemipelvis during mid-stance. These patterns may reflect impaired hip abductor control potentially related to CIPN [13,14,23]. Nonetheless, additional factors such as generalized muscular weakness, reduced physical activity, or altered neuromuscular coordination due to prolonged hospitalization or treatment-related deconditioning may also be associated with these pelvic deviations. Furthermore, the observed excessive internal–external rotation of the pelvis may indicate compensatory strategies or subtle alterations in lumbopelvic coordination. Supporting this, studies on postural control in adult survivors of childhood cancer report poorer adaptation to perturbations and greater reliance on compensatory mechanisms, reinforcing the possibility of persistent sensorimotor deficits [22]. From a clinical perspective, these frontal and horizontal plane deviations were associated with large effect sizes, indicating that these alterations are not only statistically detectable but also likely to be meaningful in terms of functional gait performance.

At the hip, reduced extension during stance was observed, which aligns with the shortened stride length and decreased propulsion observed in spatiotemporal parameters. These alterations may be related to subtle deficits in muscle activation or motor control secondary to CIPN, as well as hip flexor tightness, fatigue, or generalized deconditioning [15,25]. Increased abduction and external rotation throughout the GC may serve as compensatory adjustments to facilitate foot clearance, potentially reflecting distal motor impairments. Similar kinematic adaptations, including greater hip external rotation and reduced sagittal motion, have been documented in survivors of ALL with CIPN [16]. Importantly, the deviations observed at the hip were accompanied by large effect sizes, which suggests that these changes represent clinically meaningful disturbances in proximal joint function rather than minor or incidental variations.

In knee kinematics, the pediatric cancer survivor group demonstrated reduced flexion during loading response and mild hyperextension during mid-stance, suggesting possible impairment in eccentric quadriceps control. These deviations may also be influenced by altered proprioception, joint stiffness, or secondary musculoskeletal adaptations following chemotherapy or reduced physical activity [16,25]. Increased knee flexion during swing may represent a compensatory strategy to ensure adequate foot clearance in the presence of distal weakness or proprioceptive deficits. Similar patterns have been reported in gait of patients with neurological disorders associated with muscle weakness [26]. Finally, regarding ankle motion, pronounced alterations were observed. Both limbs exhibited excessive plantarflexion at heel strike and reduced dorsiflexion during stance, combined with diminished plantarflexion during pre-swing and limited dorsiflexion during swing. These patterns may be associated with distal weakness, partial footdrop associated with CIPN, or reduced ankle mobility and triceps surae function secondary to inactivity or treatment-related deconditioning [27,28]. Consequently, these deficits likely contribute to diminished push-off capacity and less efficient forward progression. Consistent with this interpretation, the sagittal deviations at the knee and ankle showed large effect sizes, indicating a magnitude of impairment that is relevant for clinical assessment and may warrant targeted rehabilitative intervention.

Complementing the kinematic findings, kinetic analyses revealed no significant differences at the hip or knee, whereas plantarflexor moments during terminal stance and pre-swing were reduced in the experimental group. Similarly, ground reaction force data showed a diminished first peak corresponding to initial loading and a reduced second peak during foot-off. From a clinical standpoint, these kinetic alterations were associated with large effect sizes, indicating that the magnitude of these deficits is substantial and likely to have a meaningful impact on gait performance and functional propulsion. These kinetic alterations may reinforce the hypothesis of impaired distal motor control and reduced propulsion, which could be associated with CIPN, as well as by generalized weakness or decreased neuromuscular conditioning.

The overall gait pattern observed—characterized by slower velocity, pelvic instability, reduced hip extension, knee hyperextension during stance, and distal kinetic deficits—may indicate persistent neuromuscular impairment in leukemia survivors following chemotherapy. These alterations are multifactorial, involving potential neurotoxic damage, central motor control changes, and secondary deconditioning. Importantly, these findings align with growing evidence linking cumulative exposure to vincristine and methotrexate with decreased strength, range of motion, and physical performance in long-term survivors [26].

Precise assessment using high-resolution instrumental motion analysis systems allows for the objective detection of gait impairments in pediatric cancer survivors. This enables the establishment of targeted therapeutic goals aimed at addressing the specific deficits identified in this population. The results obtained using an optoelectronic motion analysis system highlight the importance of comprehensive clinical assessments to detect balance impairments and muscle weakness in pediatric cancer survivors treated with chemotherapy. Asking patients about muscle weakness and loss of balance may help assess fall risk and should be combined with objective measures of strength and balance, such as gait observation, Romberg testing, Timed Up & Go, and grip strength testing. Similar manifestations have been reported in adult cancer survivors, where these deficits are also associated with an increased risk of falls [29].

### Limitations and Future Directions

This study has several limitations. First, the small sample size and wide age range (6–18 years) limit the generalizability of the findings, and although the sample size was relatively small. No formal priori sample size calculation was conducted due to the limited accessibility of this clinical population. Nonetheless, to contextualize the adequacy of the achieved sample, we considered what a priori calculation would typically require for this type of analysis. Using a two-tailed design for independent samples, an alpha level of 0.05, and 80% power, we adopted a large effect size (d = 0.8) as a reference value. This effect magnitude was selected because it is consistent with the average size of the meaningful differences observed across the most relevant kinematic variables in our dataset and aligns with the magnitude of gait alterations reported in prior pediatric oncology research. Under these parameters, the required sample size would have been 26 participants per group.

These constraints are common in pediatric oncology research due to the low incidence of pediatric cancers and the complexity of recruitment; however, they position this work as a pilot exploratory study. Future studies should include larger samples with narrower age ranges to better characterize developmentally specific gait alterations. Treatment protocols were heterogeneous, and we did not quantify cumulative doses of neurotoxic agents, nor specific drug exposure, all of which may influence the severity of impairments. Additionally, we did not assess physical activity levels, which may act as a confounding variable, nor did we evaluate fatigue, which may influence gait performance in pediatric cancer survivors. We also did not use a standardized clinical scale to assess peripheral neuropathy, nor did we perform electromyographic evaluations, which could have provided more direct information on neuromuscular function. Finally, only a single time-point assessment was conducted, preventing evaluation of the progression or persistence of gait alterations over time.

Future studies should include larger cohorts, stratified by treatment type and neurotoxic exposure, and incorporate longitudinal follow-up to track the development, progression, or potential recovery of gait parameters. Combining biomechanical analysis with electromyography, sensory assessments, and standardized neuropathy scales would help clarify the underlying mechanisms of the observed deficits and allow a more comprehensive understanding of long-term functional outcomes in pediatric cancer survivors.

## 5. Conclusions

Pediatric cancer survivors who have completed chemotherapy exhibit measurable gait alterations compared with healthy peers, including slower walking speed, shorter step and stride length, frontal-plane pelvic instability, altered hip, knee and ankle kinematics, and reduced ankle plantar flexor moments. Additionally, the ground reaction force corresponding to loading response was reduced in both lower limbs of the patients. These findings indicate that locomotor performance can remain affected after treatment, likely reflecting the combined impact of chemotherapy and treatment-related physical deconditioning.

## Figures and Tables

**Table 1:** Spatiotemporal parameters. Comparisons between groups.

		Mean (SD)	Median (Q1;Q3)	Min-Max	*p* for Shapiro–Wilk	*t*-Test (*p*)	Mann–Whitney (*p*)
Walking speed	Control	1.37 (0.16)	1.36 (1.22–1.49)	1.21–1.6	0.113	2.74 (0.018 *)	43 (0.017 *)
	Exp	1.15 (0.14)	1.12 (1.06 –1.24)	0.98–1.34			
Cadence	Control	117.74 (6.92)	119.86 (118.34–121.18)	102.55–122.76	0.275	1.56 (0.145)	38 (0.097)
	Exp	109.64 (11.9)	112.27 (100.07–117.91)	93.65–125.61			
Double Support	Control	0.2 (0.03)	0.19 (0.16–0.20)	0.16–0.24	0.973	1.14 (0.275)	31 (0.456)
	Exp	0.16 (0.05)	0.18 (0.12–0.19)	0.09–0.24			
Foot Off	Control	59.27 (1.01)	59.3 (58.37–60.04)	58.13–60.66	0.225	0.54 (0.602)	26 (0.902)
	Exp	58.76 (2.34)	59.6 (57.65–59.81)	55.17–61.59			
Opposite Foot Contact	Control	50.08 (0.18)	50.06 (49.95–50.23)	49.84–50.28	0.002 *	1.49 (0.163)	27 (0.805)
	Exp	47.47 (4.64)	50.16 (45.68–50.26)	39.87–50.4			
Opposite Foot Off	Control	8.64 (1.12)	8.95 (7.79–9.35)	7.06–10.18	0.215	1.39 (0.19)	33 (0.318)
	Exp	7.34 (2.21)	8.64 (6.63–9.1)	3.95–9.35			
Single Support	Control	0.42 (0.030)	0.41 (0.4–0.43)	0.39–0.48	0.219	2.27 (0.043) *	39 (0.073)
	Exp	0.32 (0.11)	0.39 (0.23–0.4)	0.18–0.43			
Step Length	Control	0.69 (0.07)	0.71 (0.65–0.74)	0.60–0.78	0.048 *	2.84 (0.015) *	42 (0.026) *
	Exp	0.59 (0.07)	0.6 (0.54–0.65)	0.5–0.66			
Step Time	Control	0.51 (0.03)	0.50 (0.49–0.50)	0.49–0.58	0.052	0.18 (0.863)	25 (0.999)
	Exp	0.51 (0.05)	0.51 (0.47–0.52)	0.45–0.61			
Step Width	Control	0.06 (0.02)	0.08 (0.06–0.08)	0.02–0.09	0.219	0.62 (0.547)	20 (0.62)
	Exp	0.07 (0.03)	0.07 (0.06–0.1)	0.03–0.11			
Stride Length	Control	1.39 (0.14)	1.41 (1.3–1.5)	1.21–1.57	0.419	2.75 (0.018 *)	41 (0.038) *
	Exp	1.22 (0.09)	1.22 (1.14–1.31)	1.11–1.33			
Stride Time	Control	1.02 (0.07)	1.01 (0.99–1.01)	0.98–1.17	0.003 *	0.54 (0.602)	17 (0.755)
	Exp	1.04 (0.09)	1.03 (1–1.06)	0.96–1.23			

Data expressed as mean (SD). Significance level: *p* < 0.05 *. Normality assessed using the Shapiro–Wilk test; between-group comparisons performed using independent two-sample t-test or Mann–Whitney *U* test as appropriate. Exp is experimental group.

**Figure A1:**
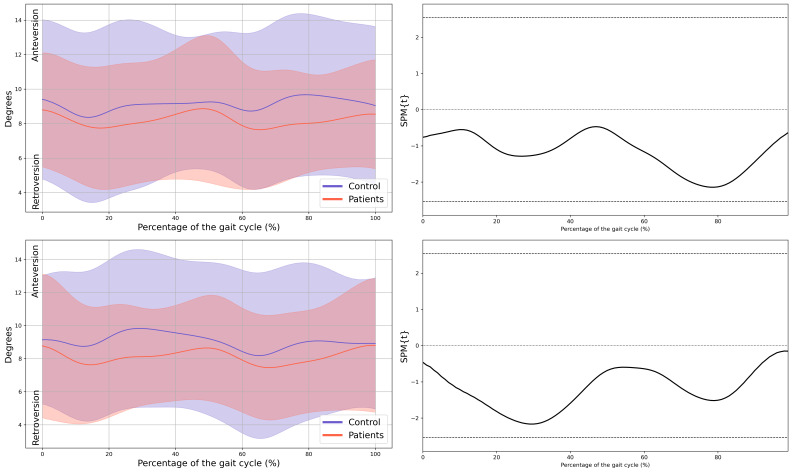
Pelvis kinematics in the sagittal plane.

**Figure A2:**
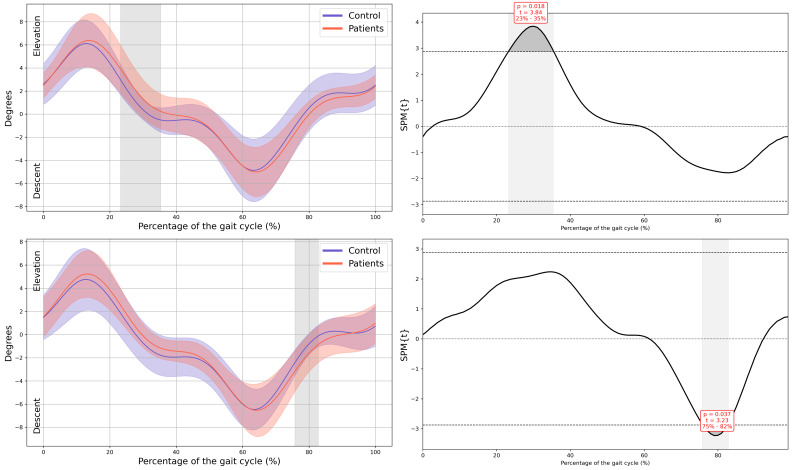
Pelvis kinematics in the frontal plane.

**Figure A3:**
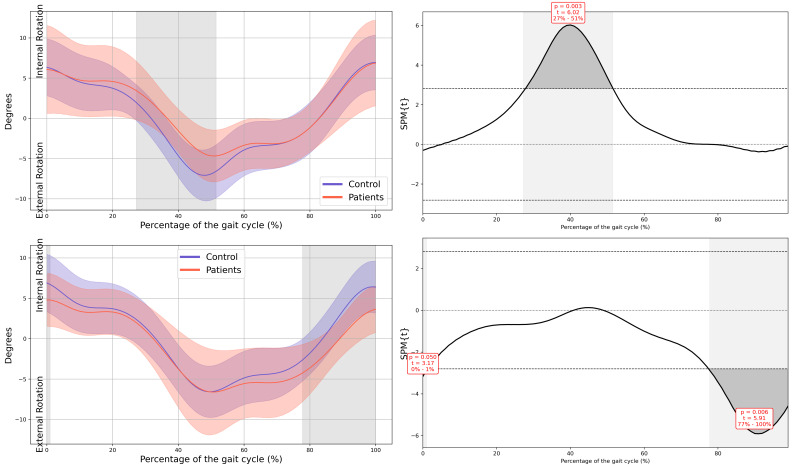
Pelvis kinematics in the horizontal plane.

**Figure A4:**
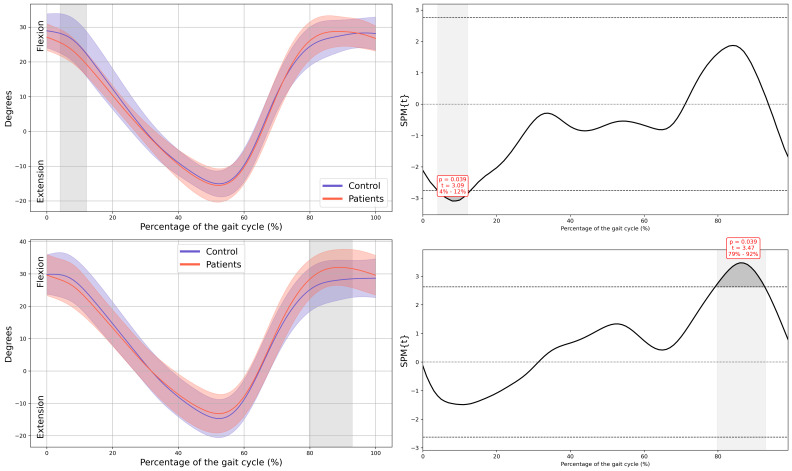
Hip kinematics in the sagittal plane.

**Figure A5:**
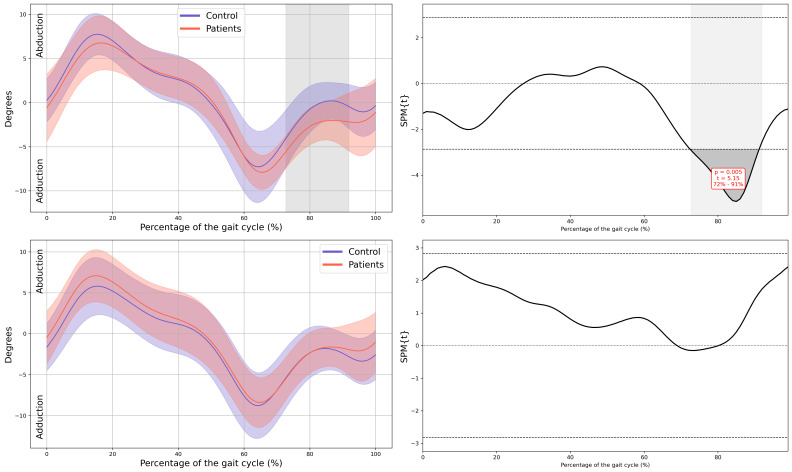
Hip kinematics in the frontal plane.

**Figure A6:**
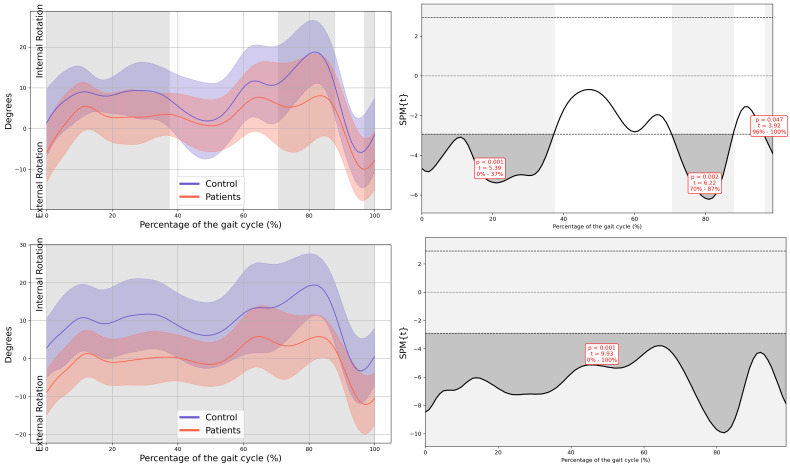
Hip kinematics in the horizontal plane.

**Figure A7:**
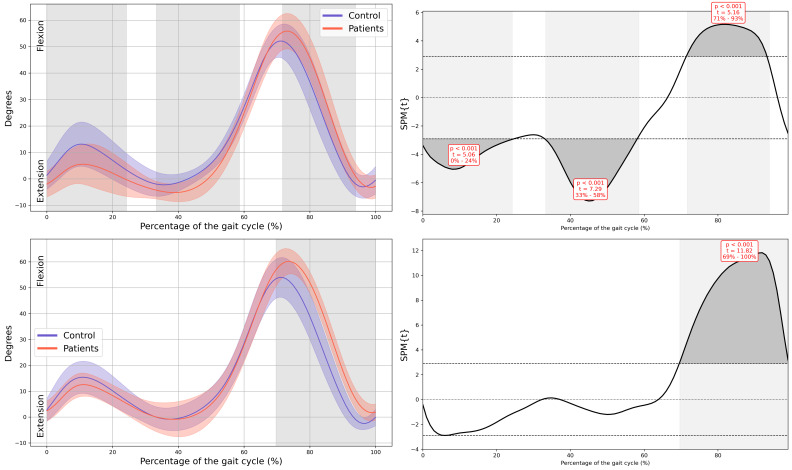
Knee kinematics in the sagittal plane.

**Figure A8:**
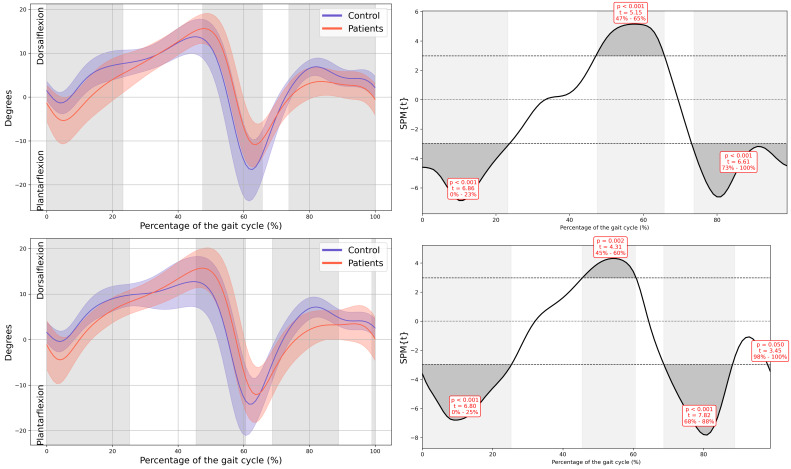
Ankle kinematics in the sagittal plane.

**Figure A9:**
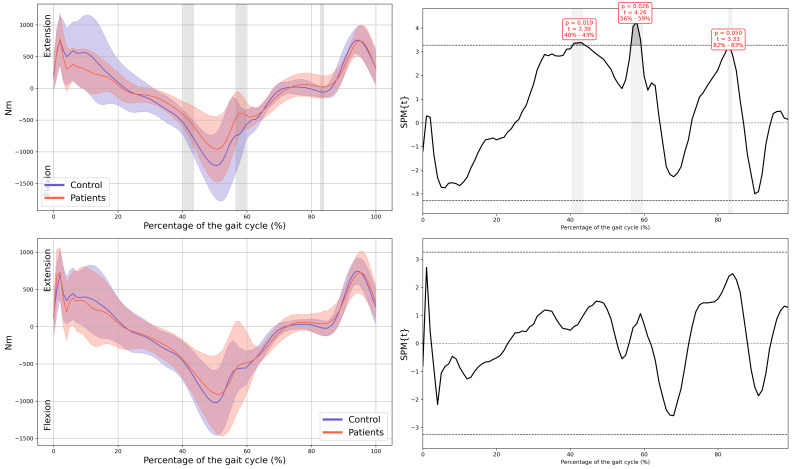
Hip internal moment in the sagittal plane.

**Figure A10:**
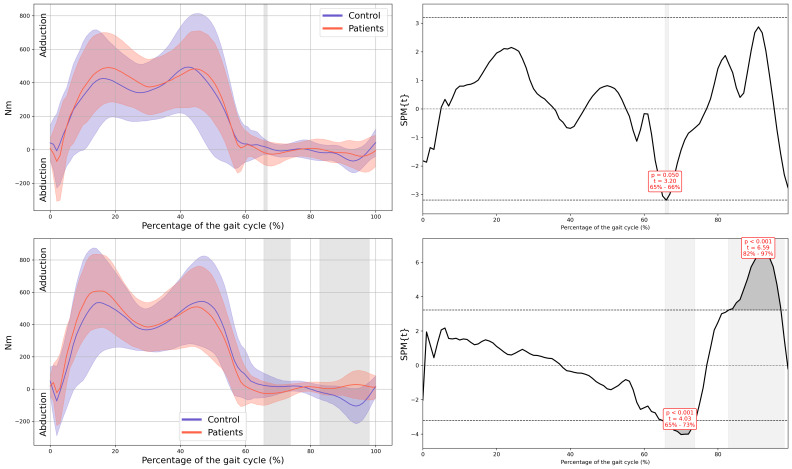
Hip internal moment in the frontal plane.

**Figure A11:**
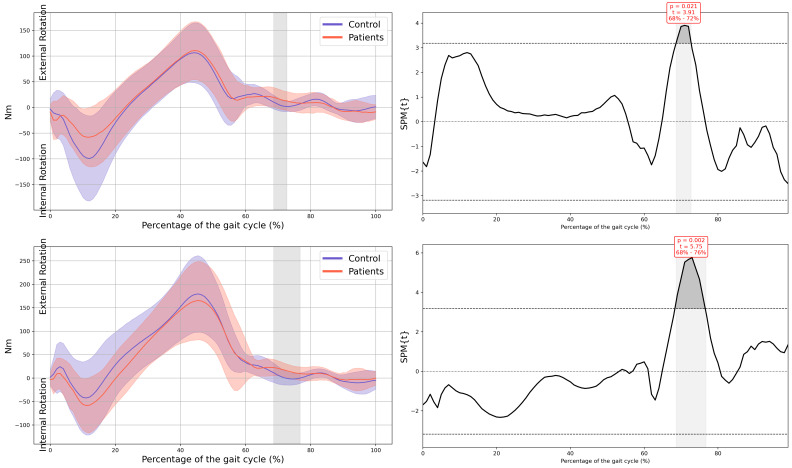
Hip internal moment in the horizontal plane.

**Figure A12:**
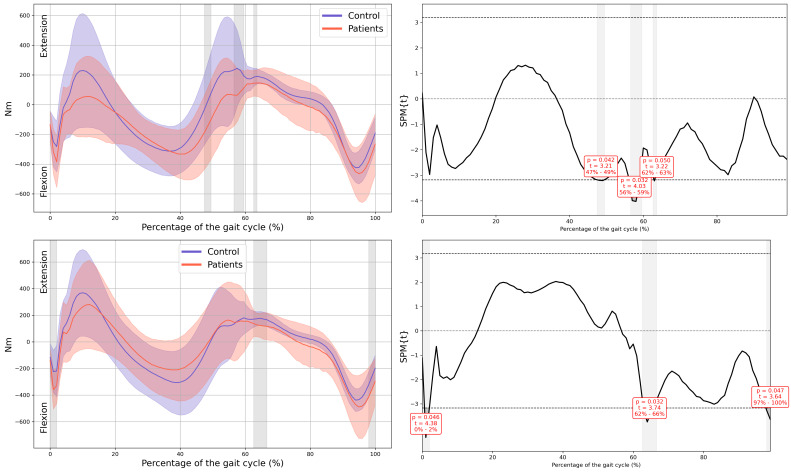
Knee internal moment in the sagittal plane.

**Figure A13:**
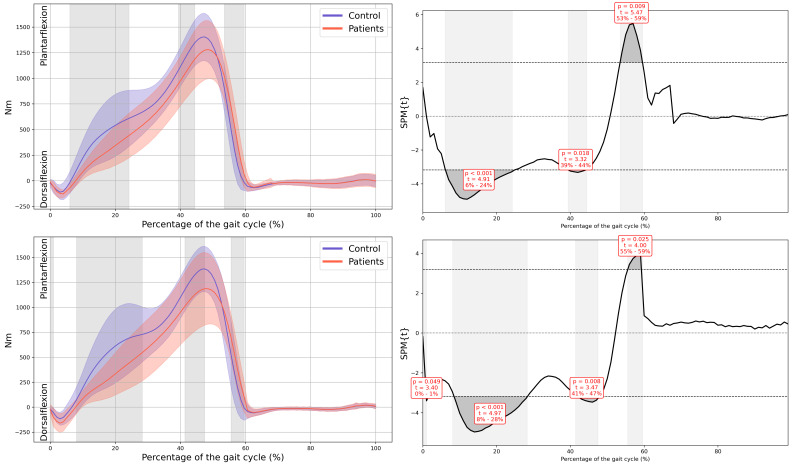
Ankle internal moment in the sagittal plane.

**Figure A14:**
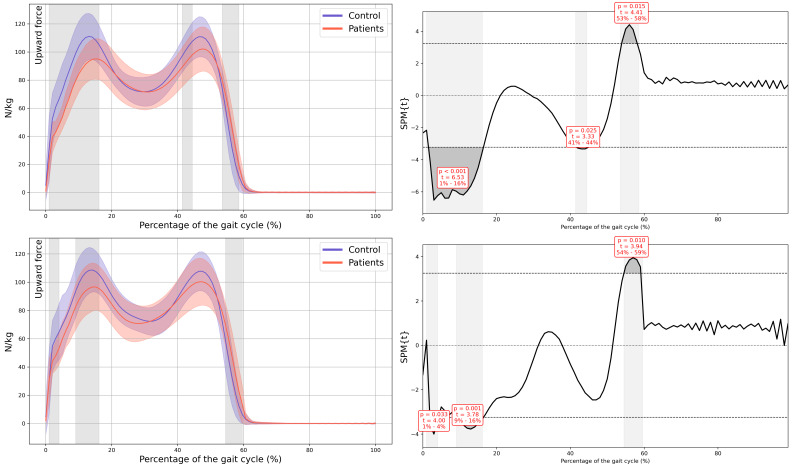
Normalized Vertical Ground Reaction Force.

## Data Availability

The data presented in this study are available on request from the corresponding author due to privacy and ethical reasons.

## Supplementary Materials

The following supporting information can be downloaded at: https://www.mdpi.com/article/10.3390/children13010096/s1. The following supporting information can be viewed in Appendix A: All graphs along with their corresponding SPM statistics, the established threshold, and the *p*-values for the segments exceeding the threshold, indicating statistically significant differences.

## Abbreviations

The following abbreviations are used in this manuscript:

ALL	Acute lymphoblastic leukemia
CIPN	Chemotherapy-induced peripheral neuropathy
CI	Confidence Interval
GC	Gait cycle
GRF	Ground reaction force
LAMBECOM	Movement Analysis, Biomechanics, Ergonomics, and Motor Control Laboratory
LAS	Less affected side
MAS	More affected side
MD	Mean Difference
SPM	Statistical Parametric Mapping

## Appendix A

### Graphs with Their Corresponding SPM Statistics

In all the figures, the first panel compares the dominant side of the healthy controls with the less affected side of the patients, whereas the second panel compares the non-dominant side with the more affected side. The *X*-axis represents the normalized gait cycle percentage from 0 to 100, and the *Y*-axis represents the corresponding kinematic or kinetic values in their standard units. The red and blue band indicates the standard deviation of the patients and healthy controls respectively. The gray band indicates the percentage of the gait cycle in which statistically significant differences between groups were observed.
