# Supplementary material for: Instrumented Assessment of Gait in Pediatric Cancer Survivors: Identifying Functional Impairments After Oncological Treatment—A Pilot Study
Source: Children (Basel). 2026 Jan 9;13(1):96. doi: 10.3390/children13010096 (PMC12840154; doi:10.3390/children13010096)
Supplement: Supplementary file 1 [file children-13-00096-s001.zip › children-3983104-supplementary.pdf]

Supplementary Materials

Left ankle angles in sagittal plane

Cluster 0:

- p-value: <0.001;
- Maximum t-statistic within the cluster: 6.861;
- Start: 0.0%;
- End: 23.2%;
- Mean Difference: -4.036;
- Lower confidence interval: -6.798;
- Upper confidence interval: -0.861.

Cluster 1:

- p-value: <0.001;
- Maximum t-statistic within the cluster: 5.146;
- Start: 47.5%;
- End: 65.7%;
- Mean Difference: 5.661;
- Lower confidence interval: 0.739;
- Upper confidence interval: 11.171.

Cluster 2:

- p-value: <0.001;
- Maximum t-statistic within the cluster: 6.610;
- Start: 73.7%;
- End: 100.0%;
- Mean Difference: -2.404;
- Lower confidence interval: -4.548;

- Upper confidence interval: -0.541.

Left ankle moment in sagittal plane

Cluster 0:

- p-value: <0.001;
- Maximum t-statistic within the cluster: 4.908;
- Start: 6.1%;
- End: 24.2%;
- Mean Difference: -163.385;
- Lower confidence interval: -306.348;
- Upper confidence interval: -19.055.

Cluster 1:

- p-value: 0.0176;
- Maximum t-statistic within the cluster: 3.324;
- Start: 39.4%;
- End: 44.4%;
- Mean Difference: -149.647;
- Lower confidence interval: -259.348;
- Upper confidence interval: -48.250.

Cluster 2:

- p-value: 0.0088;
- Maximum t-statistic within the cluster: 5.472;
- Start: 53.5%;
- End: 59.6%;
- Mean Difference: 171.728;
- Lower confidence interval: 28.748;
- Upper confidence interval: 327.918.

Left hip angles in sagittal plane

Cluster 0:

- p-value: 0.0388;
- Maximum t-statistic within the cluster: 3.091;
- Start: 4.0%;
- End: 12.1%;
- Mean Difference: -2.990;
- Lower confidence interval: -5.193;
- Upper confidence interval: -0.740.

Lef hip angles in frontal plane

Cluster 0:

- p-value: 0.0049;
- Maximum t-statistic within the cluster: 5.153;
- Start: 72.7%;
- End: 91.9%;
- Mean Difference: -1.938;
- Lower confidence interval: -3.114;
- Upper confidence interval: -0.430.

Left hip angles in horizontal plane

Cluster 0:

- p-value: <0.001;
- Maximum t-statistic within the cluster: 5.391;
- Start: 0.0%;
- End: 37.4%;

- Mean Difference: -5.408;
- Lower confidence interval: -10.162;
- Upper confidence interval: -1.288.

Cluster 1:
- p-value: 0.0022;
- Maximum t-statistic within the cluster: 6.217;
- Start: 70.7%;
- End: 87.9%;
- Mean Difference: -8.885;
- Lower confidence interval: -14.913;
- Upper confidence interval: -1.420.

Cluster 2:
- p-value: 0.0466;
- Maximum t-statistic within the cluster: 3.924;
- Start: 97.0%;
- End: 100.0%;
- Mean Difference: -5.436;
- Lower confidence interval: -9.450;
- Upper confidence interval: -1.308.

Left hip moment in sagittal plane

Cluster 0:
- p-value: 0.0186;
- Maximum t-statistic within the cluster: 3.386;
- Start: 40.4%;
- End: 43.4%;

- Mean Difference: 162.451;
- Lower confidence interval: 46.731;
- Upper confidence interval: 309.588.

Cluster 1:
- p-value: 0.0255;
- Maximum t-statistic within the cluster: 4.260;
- Start: 56.6%;
- End: 59.6%;
- Mean Difference: 265.652;
- Lower confidence interval: 72.896;
- Upper confidence interval: 503.714.

Cluster 2:
- p-value: 0.0497;
- Maximum t-statistic within the cluster: 3.330;
- Start: 82.8%;
- End: 83.8%;
- Mean Difference: 81.712;
- Lower confidence interval: 25.151;
- Upper confidence interval: 137.768.

Left hip moment in frontal plane

Cluster 0:
- p-value: 0.0500;
- Maximum t-statistic within the cluster: 3.198;
- Start: 65.7%;
- End: 66.7%;

- Mean Difference: -36.114;
- Lower confidence interval: -59.526;
- Upper confidence interval: -12.611.

Left hip moment in horizontal plane

Cluster 0:

- p-value: 0.0215;
- Maximum t-statistic within the cluster: 3.906;
- Start: 68.7%;
- End: 72.7%;
- Mean Difference: 10.688;
- Lower confidence interval: 2.767;
- Upper confidence interval: 17.538.

Left knee angles in sagittal plane

Cluster 0:

- p-value: <0.001;
- Maximum t-statistic within the cluster: 5.056;
- Start: 0.0%;
- End: 24.2%;
- Mean Difference: -5.813;
- Lower confidence interval: -10.693;
- Upper confidence interval: -1.207.

Cluster 1:

- p-value: <0.001;
- Maximum t-statistic within the cluster: 7.293;
- Start: 33.3%;

- End: 58.6%;
- Mean Difference: -3.837;
- Lower confidence interval: -6.178;
- Upper confidence interval: -0.720.

Cluster 2:
- p-value: <0.001;
- Maximum t-statistic within the cluster: 5.160;
- Start: 71.7%;
- End: 93.9%;
- Mean Difference: 6.798;
- Lower confidence interval: 0.868;
- Upper confidence interval: 12.983.

Left knee moment in sagittal plane

Cluster 0:
- p-value: 0.0422;
- Maximum t-statistic within the cluster: 3.208;
- Start: 47.5%;
- End: 49.5%;
- Mean Difference: -166.172;
- Lower confidence interval: -286.444;
- Upper confidence interval: -57.767.

Cluster 1:
- p-value: 0.0325;
- Maximum t-statistic within the cluster: 4.027;

- Start: 56.6%;
- End: 59.6%;
- Mean Difference: -143.218;
- Lower confidence interval: -279.793;
- Upper confidence interval: -32.389.

Cluster 2:
- p-value: 0.0499;
- Maximum t-statistic within the cluster: 3.218;
- Start: 62.6%;
- End: 63.6%;
- Mean Difference: -42.697;
- Lower confidence interval: -73.550;
- Upper confidence interval: -10.908.

Left normalized vertical ground reaction force

Cluster 0:
- p-value: <0.001;
- Maximum t-statistic within the cluster: 6.525;
- Start: 1.0%;
- End: 16.2%;
- Mean Difference: -16.862;
- Lower confidence interval: -26.118;
- Upper confidence interval: -0.719.

Cluster 1:
- p-value: 0.0250;

- Maximum t-statistic within the cluster: 3.330;
- Start: 41.4%;
- End: 44.4%;
- Mean Difference: -8.925;
- Lower confidence interval: -15.431;
- Upper confidence interval: -2.900.

Cluster 2:

- p-value: 0.0152;
- Maximum t-statistic within the cluster: 4.407;
- Start: 53.5%;
- End: 58.6%;
- Mean Difference: 10.380;
- Lower confidence interval: 0.410;
- Upper confidence interval: 20.535.

Left pelvis angles in frontal plane

Cluster 0:

- p-value: 0.0180;
- Maximum t-statistic within the cluster: 3.837;
- Start: 23.2%;
- End: 35.4%;
- Mean Difference: 0.916;
- Lower confidence interval: 0.252;
- Upper confidence interval: 1.581.

Left pelvis angles horizontal plane

Cluster 0:

- p-value: 0.0035;
- Maximum t-statistic within the cluster: 6.019;
- Start: 27.3%;
- End: 51.5%;
- Mean Difference: 2.520;
- Lower confidence interval: 0.357;
- Upper confidence interval: 4.309.

Right ankle angles in sagittal plane

Cluster 0:

- p-value: <0.001;
- Maximum t-statistic within the cluster: 6.796;
- Start: 0.0%;
- End: 25.3%;
- Mean Difference: -3.158;
- Lower confidence interval: -5.519;
- Upper confidence interval: -0.565.

Cluster 1:

- p-value: 0.0021;
- Maximum t-statistic within the cluster: 4.311;
- Start: 45.5%;
- End: 60.6%;
- Mean Difference: 5.460;
- Lower confidence interval: 0.823;
- Upper confidence interval: 11.150.

Cluster 2:

- p-value: <0.001;
- Maximum t-statistic within the cluster: 7.821;
- Start: 68.7%;
- End: 88.9%;
- Mean Difference: -3.535;
- Lower confidence interval: -5.954;
- Upper confidence interval: -0.489.

Cluster 3:

- p-value: 0.0496;
- Maximum t-statistic within the cluster: 3.446;
- Start: 99.0%;
- End: 100.0%;
- Mean Difference: -2.156;
- Lower confidence interval: -3.751;
- Upper confidence interval: -0.591.

Right ankle moment in sagittal Plane

Cluster 0:

- p-value: 0.0491;
- Maximum t-statistic within the cluster: 3.402;
- Start: 0.0%;
- End: 1.0%;
- Mean Difference: -24.484;
- Lower confidence interval: -74.924;
- Upper confidence interval: -15.586.

Cluster 1:

- p-value: <0.001;
- Maximum t-statistic within the cluster: 4.965;
- Start: 8.1%;
- End: 28.3%;
- Mean Difference: -213.538;
- Lower confidence interval: -403.250;
- Upper confidence interval: -20.906.

Cluster 2:

- p-value: 0.0078;
- Maximum t-statistic within the cluster: 3.468;
- Start: 41.4%;
- End: 47.5%;
- Mean Difference: -191.813;
- Lower confidence interval: -323.185;
- Upper confidence interval: -57.312.

Cluster 3:

- p-value: 0.0248;
- Maximum t-statistic within the cluster: 3.995;
- Start: 55.6%;
- End: 59.6%;
- Mean Difference: 159.623;
- Lower confidence interval: 52.131;
- Upper confidence interval: 299.647.

Right hip angles sagittal plane

Cluster 0:

- p-value: 0.0392;
- Maximum t-statistic within the cluster: 3.468;
- Start: 79.8%;
- End: 92.9%;
- Mean Difference: 3.690;
- Lower confidence interval: 0.752;
- Upper confidence interval: 6.364.

Right hip angles in horizontal plane

Cluster 0:

- p-value: <0.001;
- Maximum t-statistic within the cluster: 9.932;
- Start: 0.0%;
- End: 100.0%;
- Mean Difference: -9.910;
- Lower confidence interval: -17.070;
- Upper confidence interval: -3.563.

Right hip moment in frontal plane

Cluster 0:

- p-value: <0.001;
- Maximum t-statistic within the cluster: 4.028;
- Start: 65.7%;
- End: 73.7%;

- Mean Difference: -38.729;
- Lower confidence interval: -78.855;
- Upper confidence interval: -11.687.

Cluster 1:
- p-value: <0.001;
- Maximum t-statistic within the cluster: 6.592;
- Start: 82.8%;
- End: 98.0%;
- Mean Difference: 75.678;
- Lower confidence interval: 9.077;
- Upper confidence interval: 174.307.

Right hip moment in horizontal plane

Cluster 0:
- p-value: 0.0018;
- Maximum t-statistic within the cluster: 5.754;
- Start: 68.7%;
- End: 76.8%;
- Mean Difference: 13.861;
- Lower confidence interval: 3.248;
- Upper confidence interval: 22.870.

Right knee angles in sagittal plane

Cluster 0:
- p-value: <0.001;
- Maximum t-statistic within the cluster: 11.820;
- Start: 69.7%;

- End: 100.0%;
- Mean Difference: 9.317;
- Lower confidence interval: 0.697;
- Upper confidence interval: 16.842.

Right knee moment in sagittal plane

Cluster 0:
- p-value: 0.0460;
- Maximum t-statistic within the cluster: 4.383;
- Start: 0.0%;
- End: 2.0%;
- Mean Difference: -89.371;
- Lower confidence interval: -197.381;
- Upper confidence interval: 17.432.

Cluster 1:
- p-value: 0.0320;
- Maximum t-statistic within the cluster: 3.744;
- Start: 62.6%;
- End: 66.7%;
- Mean Difference: -46.748;
- Lower confidence interval: -81.897;
- Upper confidence interval: -2.160.

Cluster 2:
- p-value: 0.0467;
- Maximum t-statistic within the cluster: 3.641;
- Start: 98.0%;
- End: 100.0%;

- Mean Difference: -96.158;
- Lower confidence interval: -157.313;
- Upper confidence interval: -27.826.

Right normalized vertical ground reaction force

Cluster 0:

- p-value: 0.0326;
- Maximum t-statistic within the cluster: 4.004;
- Start: 1.0%;
- End: 4.0%;
- Mean Difference: -8.461;
- Lower confidence interval: -18.408;
- Upper confidence interval: 8.757.

Cluster 1:

- p-value: 0.0013;
- Maximum t-statistic within the cluster: 3.776;
- Start: 9.1%;
- End: 16.2%;
- Mean Difference: -11.738;
- Lower confidence interval: -19.680;
- Upper confidence interval: -3.943.

Cluster 2:

- p-value: 0.0100;
- Maximum t-statistic within the cluster: 3.945;
- Start: 54.5%;

- End: 59.6%;
- Mean Difference: 11.873;
- Lower confidence interval: 3.149;
- Upper confidence interval: 22.381.

Right pelvis angles in frontal plane

Cluster 0:

- p-value: 0.0366;
- Maximum t-statistic within the cluster: 3.229;
- Start: 75.8%;
- End: 82.8%;
- Mean Difference: -0.775;
- Lower confidence interval: -1.305;
- Upper confidence interval: -0.199.

Right pelvis angles in horizontal plane

Cluster 0:

- p-value: 0.0496;
- Maximum t-statistic within the cluster: 3.174;
- Start: 0.0%;
- End: 1.0%;
- Mean Difference: -2.015;
- Lower confidence interval: -3.423;
- Upper confidence interval: -0.593.

Cluster 1:

- p-value: 0.0063;

- Maximum t-statistic within the cluster: 5.912;
- Start: 77.8%;
- End: 100.0%;
- Mean Difference: -2.629;
- Lower confidence interval: -4.370;
- Upper confidence interval: -0.425.
